# Endoscopic endonasal transclival petroclival meningioma resection

**DOI:** 10.3171/2022.1.FOCVID21209

**Published:** 2022-04-01

**Authors:** Stephen T. Magill, Ben G. McGahan, Ricardo L. Carrau, Daniel M. Prevedello

**Affiliations:** 1 Department of Neurological Surgery, The Ohio State University Medical Center, Columbus, Ohio;; 2 Department of Otolaryngology, The Ohio State University Medical Center, Columbus, Ohio; and; 3 Department of Neurological Surgery, Northwestern University, Feinberg School of Medicine, Chicago, Illinois

**Keywords:** petroclival, meningioma, skull base, pituitary, transclival, endoscopic endonasal, neuroendoscopy

## Abstract

Petroclival meningiomas are surgically challenging due to the surrounding neurovascular structures. Petroclival meningiomas located inferior to the oculomotor nerve and superior or medial to the abducens nerve are ideal for an endoscopic endonasal transclival approach because this prevents the need to work across cranial nerves, limiting operative risk. The authors present a case of a 45-year-old woman with a growing petroclival meningioma that was distorting the pons. In the video they demonstrate the technique and discuss nuances of petroclival meningioma resection via an endoscopic endonasal transclival approach with posterior clinoidectomy.

The video can be found here: https://stream.cadmore.media/r10.3171/2022.1.FOCVID21209

## Transcript

This video demonstrates an endoscopic endonasal transclival approach for resection of a petroclival meningioma.

### 0:27 History.

Patient was a 45-year-old lady who presented with one episode of whole-body numbness, that fully recovered. An MRI was performed to rule out a stroke that identified the presence of a dorsum clival petroclival meningioma. The initial recommendation was observation, and the patient came back 6 months later with an MRI demonstrating increase on the size of the tumor.

### 0:54 Imaging.

The initial presentation had a small meningioma not causing major mass effect and 6 months later showed further distortion of the ventral aspect of the pons and an increase of 5 mm in a lateral perspective of the tumor and 3 mm in the anterior-posterior dimension. In the sagittal MRI one could see that the tumor was protruding above the level of the dorsum sellae invading the inferior aspect of the interpeduncular fossa immediately below the level of the left third cranial nerve. The tumor was also immediately above the position of the left sixth cranial nerve. Due to this specific peculiar position, we indicated an endoscopic endonasal transclival transdorsum approach to resect this tumor that was specifically located below the left third cranial nerve and above the left sixth cranial nerve.^1–5^

### 1:54 Operative Video.

An endoscopic endonasal approach was performed and a nasal septal flap was elevated on the right side of the nose. The anterior wall of the sphenoid was drilled, and then all the septations of the sphenoid were drilled as well as the face of the sella. We then started drilling the clival bone immediately medial to the left internal carotid artery. The dura of the sella and the face of the cavernous sinus was exposed, and the dura was cut in the periosteal layer to allow for an interdura dissection and transposition of the pituitary gland. That maneuver allowed us to expose the posterior clinoid on the left side, and a branch of the posterior meningohypophyseal trunk was dissected, and this very small vessel was attached to the posterior clinoid—most likely was the dorsal meningeal that we dissected away from the posterior clinoid—allowing for a resection of the medial aspect of the carotid canal on the left side and full exposure of the dura located posteriorly. Once the bony structures were removed, we opened the dura very inferiorly and we inspected the prepontine cistern identifying the sixth cranial nerve immediately inferior to the tumor on the left side, as well as branches of the basilar artery and basilar artery itself. We removed the dura progressively superiorly, and we use bipolar to coagulate the basilar plexus and we were able then to go in around the medial aspect of the tumor, following the basilar artery safely from inferior to superior. As we dissected laterally, we performed all the maneuvers with direct visualization of the left sixth cranial nerve inferiorly, and superiorly we identified the position of the arachnoid and also the location of the left third cranial nerve, with the visualization of both those cranial nerves and the full understanding that the insertion of the tumor was located between the third and the sixth cranial nerve. We used a 90° curette to remove the insertion of the tumor that was immediately lateral and located immediately posterior to the left carotid artery. With this insertion we were able to mobilize it to more safely and protecting the left sixth and the left third cranial nerve. It was a complete resection of the tumor, and further visualization inspection with a 45° endoscope proved to be a complete resection of the tumor with no residual on the lateral aspect. The reconstruction was performed with a collagen matrix, followed by what we call a soft gasket sealed with Gelfoam inside, fat graft, and utilization of the nasal septal flap for full reconstruction of the skull base.

### 5:00 Postoperative Imaging.

The postoperative imaging shows the presence of the fat graft in the clival recess as well as the nasal septal flap covering the entire skull base defect and complete resection of the tumor without any complications.

### 5:16 Postoperative Course.

After surgery, patient did very well and was discharged on the 3rd day after surgery without any signs of leakage. Six days after surgery, patient came back with signs of hyponatremia that was treated and patient was discharged, doing very well with no neurological deficits. No CSF leakage and no complications. Patient was recently seen 6 months after surgery. The postoperative MRI confirming a total resection of the tumor. No residual and no recurrence, and the patient is doing very well.

## Disclosures

Dr. Prevedello reports personal fees from Storz, Stryker, and Integra LifeSciences; other from Mizuho, KLS Martin, and ACE Medical; grants from Integra LifeSciences; and personal fees from BK Medical outside the submitted work.

## Author Contributions

Primary surgeon: Carrau, Prevedello. Assistant surgeon: Magill. Editing and drafting the video and abstract: Magill, McGahan, Prevedello. Critically revising the work: all authors. Reviewed submitted version of the work: all authors. Supervision: Carrau, Prevedello.
